# Progression to Dementia in Very Late‐Onset Schizophrenia‐Like Psychosis Stratified by Alzheimer's Disease and Lewy Body Disease Biomarkers: A Retrospective Cohort Study

**DOI:** 10.1111/psyg.70205

**Published:** 2026-08-10

**Authors:** Yuto Satake, Hideki Kanemoto, Daiki Taomoto, Kayo Takeda, Shigeki Katakami, Matasaburo Kobayashi, Keiko Matsunaga, Kayako Isohashi, Takashi Suehiro, Kenji Yoshiyama, Manabu Ikeda

**Affiliations:** ^1^ Department of Psychiatry The University of Osaka Graduate School of Medicine Suita Osaka Japan; ^2^ Health and Counseling Center The University of Osaka Toyonaka Osaka Japan; ^3^ Department of Psychiatry Osaka Psychiatric Medical Center Hirakata Osaka Japan; ^4^ Department of Nuclear Medicine Osaka International Cancer Institute Chuo‐ku Osaka Japan; ^5^ Department of Radiology The University of Osaka Graduate School of Medicine Suita Osaka Japan; ^6^ Brain and Mind Future Medical Center Osaka Psychiatric Medical Center Hirakata Osaka Japan

**Keywords:** Alzheimer's disease, biomarker, dementia, late‐onset psychosis, Lewy body disease, very late‐onset schizophrenia‐like psychosis

## Abstract

**Background:**

Very late‐onset schizophrenia‐like psychosis (VLOSLP) is clinically heterogeneous, and its relationship with dementia‐related neurodegenerative disease remains unresolved. We examined whether Alzheimer's disease (AD) and Lewy body disease (LBD) biomarker status were associated with dementia progression in VLOSLP.

**Methods:**

We retrospectively identified patients who visited the University of Osaka Hospital between January 2018 and December 2023 and met criteria for VLOSLP. Twenty‐two participants with AD and/or LBD biomarker data and at least one follow‐up assessment within 775 days were classified as biomarker‐negative (BMs‐neg; *n* = 7) or biomarker‐positive (BMs‐pos; *n* = 15). Group comparisons were performed using Mann–Whitney *U* tests and Fisher's exact tests.

**Results:**

The BMs‐pos group showed older onset age and lower memory scores than the BMs‐neg group. Dementia progression was more frequent in the BMs‐pos group than in the BMs‐neg group, although the difference was not statistically significant (8/15 [53.3%] vs. 1/7 [14.3%]; *p* = 0.165; odds ratio 6.31; 95% CI 0.55–353.18). Five of eight participants with AD biomarker positivity progressed to AD dementia. Three of seven participants with LBD biomarker positivity progressed to dementia, including two diagnosed with dementia with Lewy bodies. Follow‐up MMSE, CDR, and CDR‐SB scores differed significantly between groups.

**Conclusions:**

AD and/or LBD biomarker‐positive VLOSLP may be associated with greater dementia progression and cognitive decline, although findings should be interpreted cautiously given the small sample size and retrospective design. These results support the clinical value of considering neurodegenerative biomarkers when evaluating the prognosis and underlying pathology of VLOSLP.

## 1 Background

Late‐onset psychotic disorder is a clinical entity which is not defined by existing operational criteria. However, it has been attracting attention from geriatric psychiatrists for many years. Because it has some features that differ from the average‐onset schizophrenia spectrum disorders, such as a clear female preponderance and lack of negative symptoms [1, 2], it has been recognised as a possible distinct disorder from the average‐onset ones, and several terms have been proposed. Various terms have been used, including late paraphrenia [3], Kontaktmangelparanoid [4], and very late‐onset schizophrenia‐like psychosis (VLOSLP) [5]. Although its proportion among diseases presenting late‐onset psychotic symptoms is limited [6], the number of patients who may be diagnosed with late‐onset psychotic disorder is not small [7, 8]. It is becoming an ever more important concept in countries where the population is ageing, and the number of older people living alone is increasing, like Japan.

When we discuss this entity, it is essential to consider its relationship with dementia‐related neurodegenerative diseases [9]. VLOSLP, the latest name of this disease group, is the current umbrella term defining late‐onset psychotic disorders with an age of onset after 60 years. Although the criteria proposed by Howard et al. define psychosis as not explained by organic diseases or affective disorders, they did not show an explicit method to exclude an organic aetiology of psychosis in cases where there is a latent neurodegenerative disease [5]. At that time, biomarkers such as amyloid positron emission tomography (PET) were not yet available. Most previous studies on VLOSLP adopted Mini‐Mental State Examination (MMSE) scores (mostly more than 23 or 24) and clinicians' judgments [10, 11] to exclude neurodegenerative diseases in patients with VLOSLP cross‐sectionally. Therefore, we previously tried to investigate whether patients with VLOSLP showed positivity in neurodegenerative biomarkers, and some had positive results for Alzheimer's disease (AD) biomarkers [12] and Lewy body disease (LBD) biomarkers [13]. The biomarker‐positive patients with VLOSLP had several differences in characteristics, which were thought to derive from each neuropathology. However, we did not conclude what those results meant. Several reported that late‐onset psychotic disorders had a higher conversion rate to dementia in several years after their diagnosis [8, 14], although some did not [15]. Late‐onset psychosis may be prodrome, risk factor or complication of neurocognitive disorders [16, 17]. In the field of dementia with Lewy bodies (DLB), increasing attention has been paid to prodromal presentations, including MCI‐onset, delirium‐onset, and psychiatric‐onset presentations [17]. Previous studies have highlighted that hallucinations, REM sleep behaviour disorder, autonomic symptoms, hyposmia, motor symptoms, and other neuropsychiatric features may appear before overt dementia in prodromal DLB [18, 19].

Here, we conducted a retrospective observational cohort study of VLOSLP classified with the positivity of AD or LBD biomarkers to elucidate the relationship between those biomarkers' results and prognosis of VLOSLP. This study may deepen our understanding of this complex and heterogeneous disease area.

## 2 Methods

### 2.1 Participants

Participants were enrolled from the database of consecutive patients who had visited the neuropsychology clinic in the Department of Psychiatry at the University of Osaka Hospital for the first time between January 2018 and December 2023. The study excluded cases lacking data on the Mini‐Mental State Examination (MMSE), Clinical Dementia Rating (CDR), and either the Neuropsychiatric Inventory (NPI) or the Brief Psychiatric Rating Scale (BPRS). We defined VLOSLP according to the criteria adopted by previous studies [12, 13]. Its criteria were as follows: (1) onset of delusions and/or hallucinations at the age of 60 years or more; (2) existence of delusions and/or hallucinations at the first visit; (3) MMSE score ≥ 24; (4) a global CDR score < 1; (5) without the diagnosis of affective disorder; (6) without abnormal localised findings on magnetic resonance imaging (MRI) scans, indicating cerebrovascular disease or brain tumour; and (7) without a concurrent organic disease that could cause psychosis or physical symptoms suggestive of the disease. The existence of psychosis (delusions and/or hallucinations) was defined with either (i) being scored on the NPI subitems of delusions and/or hallucinations or (ii) psychosis confirmed by medical history and BPRS ≥ 30. Thereafter, we extracted the VLOSLP patients who had at least one follow‐up assessment within 2 years and 1.5 months (775 days) of the initial assessment. Finally, we selected two types of participants from the patients above: (1) both AD and LBD biomarker‐negative (BMs‐neg) and (2) AD and/or LBD biomarker‐positive (BMs‐pos), based on the reported diagnostic performance of the biomarkers used in this study [20, 21, 22, 23]. This classification reflected biomarker status around the time of assessment and was not intended to establish the complete absence or presence of underlying AD or LBD pathology. VLOSLP was used as a broad descriptive clinical construct encompassing late‐onset, delusion‐predominant psychotic presentations, with historical continuity with concepts such as late paraphrenia [5]. Participants were not further subclassified according to specific DSM or ICD psychotic disorder diagnoses; therefore, some may also have met criteria for late‐onset delusional disorder.

### 2.2 Data Collection

We extracted all the clinical data for this study from the hospital's electronic health records. In our clinic, we routinely conduct interviews about demographic data and medical history, neurological examination, standard neuropsychological examinations, standard laboratory tests, and brain neuroimaging for the initial clinical assessment. General cognition was assessed using MMSE. Memory was assessed using Wechsler Memory Scale‐Revised (WMS‐R) Logical Memory (LM) immediate recall (I) and delayed recall (II) subtests. Dementia severity was evaluated using CDR. These were conducted by neuropsychiatrists and neuropsychologists specialising in geriatric psychiatry. Neuropsychiatrists assessed neuropsychiatric symptoms using the NPI and BPRS. Clinical assessments were not conducted at uniform intervals. In this study, the latest results of assessments carried out during 775 days from the day of the first examination, which means 2 years and 1.5 months, were considered the follow‐up assessment results.

Progression to dementia was defined as a global CDR score of 1 or higher at follow‐up. The CDR rating was determined by multiple geriatric psychiatrists based on clinical interviews, cognitive assessments, and information on everyday functioning obtained from patients and informants. Dementia subtype diagnoses, including AD dementia and DLB, were made according to established diagnostic criteria through clinical chart review. Biomarker results were not used to define the primary outcome of dementia progression, which was based on the global CDR score.

### 2.3 Fluid and Imaging Biomarkers Assessment

We collected the results of amyloid positron emission tomography (PET), cerebrospinal fluid (CSF) testing, 2β‐carbomethoxy‐3β‐(4‐iodophenyl)‐N‐(3‐fluoropropyl) nortropane (FP‐CIT) single‐photon emission computed tomography (SPECT), and metaiodobenzylguanidine (MIBG) myocardial scintigraphy performed within 3 years from the first visit for cross‐sectional comparison. If the amyloid PET and CSF p‐tau were not concordant, the amyloid PET result was used as the AD biomarker result; if FP‐CIT SPECT and MIBG myocardial scintigraphy did not match, either positive result was used as the LBD biomarker result. We used ^18^F‐Florbetapir and ^18^F‐Flutemetamol amyloid PET results and CSF p‐tau levels as AD biomarkers. Amyloid PET positivity was determined by experienced nuclear medicine radiologists. The CSF collection and p‐tau measuring methods followed our previously written method [12]. Only the samples collected after April 2022 were analysed by SRL Inc. using a fully automated chemiluminescent enzyme immunoassay Lumipulse (Fujirebio, Tokyo). The reference range set by the company was 21.5–59.0 pg/mL, and samples with a value of 59.0 or higher were judged to have AD pathology.

### 2.4 Statistical Analyses and Chart Review

First, we compared the demographic characteristics, MMSE and CDR between the participants and other VLOSLP patients without either follow‐up data or biomarker data sufficient to qualify as participants. This was to check for potential selection bias due to high drop‐out due to a lack of biomarker data and follow‐up. Second, the demographics and duration between the first and the follow‐up assessments were compared between the groups. The scores from cognitive tests such as MMSE, WMS‐R LM and CDR at both the initial and the follow‐up assessments were also compared between the groups. Mann–Whitney *U* and Fisher's exact tests were used. As the primary outcome, we compared progression rates to dementia at follow‐up between the BMs‐neg and BMs‐pos groups using Fisher's exact test. Odds ratios and 95% confidence intervals were estimated using the conditional maximum likelihood method implemented in Fisher's exact test. No formal sample size calculation was performed because this was an exploratory retrospective study of a rare clinical population. All eligible consecutive cases with available biomarker and follow‐up data during the study period were included. For Mann–Whitney *U* tests, effect size *r* was calculated as the standardised *z* statistic divided by the square root of the number of observations.

This study allowed missing data on scales other than MMSE and CDR when statistical tests were performed. All statistical analyses adopted a two‐tailed *p*‐value under 0.05 as having statistical significance. Statistical analyses were performed using RStudio (version 2024.09.1+394; RStudio, PBC, Boston, MA, USA) with R (version 4.4.2, 2024‐10‐31). Complementarily, a detailed chart review of psychotic symptoms was conducted in all cases.

## 3 Results

### 3.1 Enrollment and Patients' Baseline Characteristics

**FIGURE 1:**
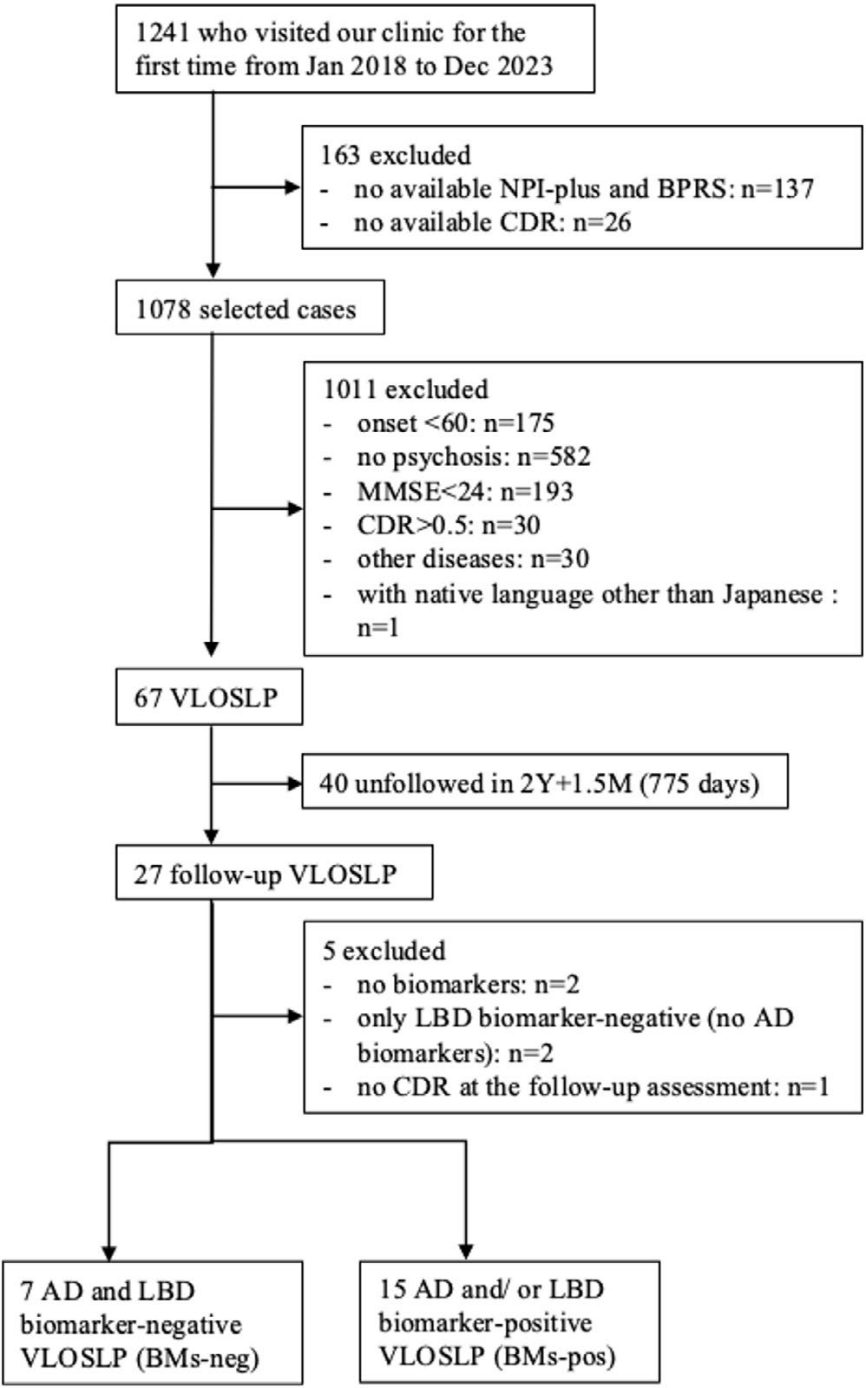
Enrollment flowchart. AD, Alzheimer's disease; BPRS, brief psychiatric rating scale; CDR, clinical dementia rating; LBD, Lewy body disease; MMSE, mini‐mental state examination; NPI‐plus, neuropsychiatric inventory‐plus; VLOSLP, very‐late‐onset schizophrenia‐like psychosis.

We enrolled the participants from 1241 patients who visited our neuropsychological clinic from January 2018 to December 2023. Out of 67 VLOSLP patients, 22 were included in this study (Figure 1).

The demographic data of the participants and the unenrolled VLOSLP patients did not show any significant differences (Table S1). There were 7 participants in the BMs‐neg group and 15 in the BMs‐pos group. At the initial assessment, the BMs‐pos group had a significantly later age at onset and significantly lower WMS‐R LM I and II scores than the BMs‐neg group. The BMs‐pos group was also older and had lower baseline MMSE scores, although these differences did not reach statistical significance (Table 1). Table S2 summarises biomarker findings, core clinical symptoms of DLB, psychotic symptom categories, and dementia progression among the included participants.

**TABLE 1:** Demographic characteristics and cognitive and dementia severity measures.

		BMs‐neg (*n* = 7)	BMs‐pos (*n* = 15)	*U*	*p*	*r*
Age, years		72 (67.5–81.5)	84 (75.5–86)	24.5	0.052	0.42
Sex, *n* of female		7 (100%)	12 (80%)	NA	0.523	NA
Onset age, years		65 (63–67)	82 (70.5–83.5)	13.5	**0.006**	0.59
Education, years		14 (12–14)	12 (12–13)	64	0.412	0.17
Time to the follow‐up, days		474 (426–510)	488 (379.5–629.5)	49.5	0.860	0.05
MMSE	Initial	29 (26–29.5)	26 (24.5–27.5)	74.5	0.125	0.33
	Follow‐up	27 (26–29)	24 (23–26)	83.5	**0.030**	0.47
WMS‐R LM I	Initial	17 (15–20)	8.5 (6–13.25)	82	**0.015**	0.54
	Follow‐up	19 (14.5–21.5)	9 (5–13)	82.5	**0.004**	0.66
WMS‐R LM II	Initial	14 (13–15)	4.5 (1–6)	96.5	**< 0.001**	0.77
	Follow‐up	12 (10.5–18)	2 (1–7)	81	**0.005**	0.63
CDR	Initial	0.5 (0.5–0.5)	0.5 (0.5–0.5)	48.5	0.621	0.06
	Follow‐up	0.5 (0–0.5)	1 (0.5–1)	24	**0.035**	0.43
CDR‐SB	Initial	2 (1.75–2.5)	2.5 (1.5–3.25)	42	0.475	0.16
	Follow‐up	1 (0.5–1.5)	4.5 (2.5–5)	15.5	**0.010**	0.56

*Note:* Data are presented as median (interquartile range) or *n* (%). The Mann–Whitney *U* test was used for continuous and ordinal variables, and Fisher's exact test was applied for categorical variables. A *p*‐value less than 0.05 was considered statistically significant in a two‐tailed test and is shown in bold. The number of observations varied across variables due to missing data.

Abbreviations: BMs‐neg, biomarkers‐negative group; BMs‐pos, AD and/or LBD biomarkers‐positive group; CDR, clinical dementia rating; CDR‐SB, clinical dementia rating sum of boxes; LM I, logical memory I (immediate recall); LM II, logical memory II (delayed recall); MMSE, mini‐mental state examination; NA, not applicable; WMS‐R, Wechsler memory scale‐revised.

### 3.2 The Rate of Progression to Dementia

A greater proportion of patients in the BMs‐pos group converted compared to the BMs‐neg group (8/15 vs. 1/7). Fisher's exact test did not reach statistical significance (*p* = 0.165), with an odds ratio of 6.31 (95% CI: 0.55–353.18). At the follow‐up assessment, among the eight participants with AD biomarker positivity, five progressed to dementia and were diagnosed with AD dementia [24]; among the seven participants with LBD biomarker positivity, three progressed to dementia; two were diagnosed with DLB [25], and one was diagnosed with possible idiopathic normal pressure hydrocephalus (iNPH) [26]. The single patient in the BMs‐neg group who progressed to dementia was not assigned a specific dementia subtype because her functional deficits were affected by her severe apathy rather than cognitive deterioration. Details of the type of dementia each patient progressed to are provided in Table S2.

### 3.3 Cognitive and Dementia Severity Changes

Table 1 also shows the results of the cognitive tests and dementia severity scales at the follow‐up assessment point. Although MMSE, CDR, and CDR‐SB scores did not significantly differ between the groups at the initial assessment, significant differences were observed at the follow‐up assessment in MMSE (*p* = 0.030, *r* = 0.47), CDR (*p* = 0.035, *r* = 0.43), and CDR‐SB scores (*p* = 0.010, *r* = 0.56).

### 3.4 Illustrative Clinical Courses

Two illustrative clinical courses are briefly described. The first participant was a 68‐year‐old woman who developed persecutory delusions, including beliefs that neighbours entered her home, moved her belongings, followed her outside, and directed lights towards her. At the initial assessment, her MMSE score was 28 and global CDR was 0.5. Amyloid PET and CSF p‐tau were positive. Her MMSE score declined to 25 after 1 year and to 23 after 2 years. By the 2‐year follow‐up, recent memory impairment had become evident in daily life, and she was no longer able to shop or cook independently. Her global CDR score had increased to 1, and she was diagnosed with AD dementia. This case has been reported in detail previously [27].

The second participant was a 74‐year‐old woman who developed probable REM sleep behaviour disorder approximately 1 year before her initial visit. Later that year, she underwent surgery for colon cancer and developed postoperative delirium. Following discharge, cognitive fluctuations became more apparent, and she began searching for her belongings more frequently. She subsequently developed delusions that others pitied her because of her illness, that people around her regarded her as poor, and that a person living upstairs was stalking her. At the initial assessment, her MMSE score was 25 and her global CDR score was 0.5. One year later, it had declined to 21, and her global CDR score had increased to 1. FP‐CIT SPECT showed a positive result, and she was subsequently diagnosed with DLB.

## 4 Discussion

In the present study, we retrospectively investigated the longitudinal clinical course of patients with VLOSLP, who were classified into two groups according to the results of AD and LBD biomarkers. We compared the progression rates to dementia within a prespecified follow‐up window of 775 days and the clinical cognitive data at both the initial and follow‐up assessment points.

We did not identify a statistically significant difference in progression to dementia between the two groups. However, only 14.3% (1/7) of participants in the BMs‐neg group developed dementia, compared to 53.3% (8/15) in the BMs‐pos group within approximately 2 years. We believe that the lack of significant difference was largely a consequence of the small number of participants studied and a lack of statistical power. Several epidemiological cohort studies reported a higher conversion rate to dementia in patients with VLOSLP [8, 28, 29]. Stafford et al. reported the highest conversion rate in the first follow‐up year after the first visit to medical facilities [8]. These previous findings are consistent with our observation of substantial cognitive differences and a higher, although not statistically significant, conversion proportion in the BMs‐pos group within approximately 2 years. Brodaty et al. reported that late‐onset schizophrenia patients, including VLOSLP, progressed to dementia more than the healthy control in a five‐year follow‐up [14]. Although the difference in conversion rates did not reach statistical significance in the present study, the higher proportion of dementia progression in the BMs‐pos group and the significant differences in follow‐up cognitive and dementia severity measures are compatible with these reports.

The relatively low frequency of dementia progression in the BMs‐neg group should be interpreted cautiously. Nagendra and Snowdon and Takeda et al. reported that many patients with late‐onset delusional disorder did not progress to dementia over several years, suggesting that some participants in the BMs‐neg group may have had a relatively stable delusional‐disorder presentation [30, 31]. However, Takeda et al. also identified delusion‐onset late‐life psychiatric presentations among patients with autopsy‐confirmed Lewy body disease [31]. In addition, Fujishiro et al. reported a case of psychiatric‐onset prodromal DLB in which proposed LBD biomarkers changed longitudinally, suggesting that biomarker abnormalities may become apparent only during subsequent follow‐up [32]. Therefore, a negative LBD biomarker result at a single assessment does not completely exclude underlying LBD. The BMs‐neg group may have included both patients with relatively stable late‐onset delusional disorder and patients with early LBD that were not detected by the available biomarker assessments.

Among the eight participants with AD biomarker positivity, five progressed to AD dementia. Among the seven participants with LBD biomarker positivity, three progressed to dementia, including two diagnosed with DLB and one diagnosed with possible iNPH. These LBD‐related findings extend previous work on prodromal DLB and MCI‐LB by applying this framework to a clinical population diagnosed with VLOSLP, in whom psychosis was the dominant presenting syndrome before overt dementia. Donaghy and colleagues have shown that neuropsychiatric symptoms, including hallucinations and delusions, can contribute to the clinical characterisation of MCI‐LB and its differentiation from MCI‐AD [33, 34]. Our study further suggests that VLOSLP may include cases that are better conceptualised as psychiatric‐onset prodromal Lewy body disease, particularly when LBD biomarkers or core clinical features are present. VLOSLP should be considered a clinically defined but biologically heterogeneous syndrome.

These results suggest that biomarker results may help predict dementia phenotype to some extent. However, AD is also often comorbid with many neurodegenerative diseases [35], and there have been warnings about the hasty assumption that AD explains a patient's symptoms based on the fact that they are positive for AD biomarkers [36]. The possible copathology needs to be considered in LBD as well. Furthermore, FP‐CIT SPECT can also show abnormalities in other neurodegenerative diseases that present with parkinsonism, such as frontotemporal dementia, corticobasal degeneration disease, and supranuclear palsy [37]. It has been reported that some iNPH patients without concomitant α‐synuclein pathology also showed abnormalities in FP‐CIT SPECT [38]. Gibson et al. reported that the core symptoms of DLB are highly reliable predictors of future DLB diagnoses in VLOSLP [39]. Considering that our patients who developed iNPH in this study did not show any core symptoms, the patient may not have had underlying LBD. It is essential to consider both comorbidity and false positives when interpreting positive biomarker results in the VLOSLP stage.

Although overt dementia was excluded using MMSE and global CDR criteria, these criteria could not completely exclude very early or prodromal AD. In our previous case report and cross‐sectional study, we raised the possibility that some patients clinically diagnosed with VLOSLP may represent prodromal AD or MCI due to AD with psychosis [12, 27]. The present longitudinal findings, in which 5 of 8 AD biomarker‐positive participants progressed to AD dementia, are consistent with this interpretation. However, this progression may partly reflect the expected clinical course of AD that was already present at baseline, including atypical phenotypes such as hippocampal‐sparing AD. Therefore, the present study cannot determine whether psychosis was an early manifestation of AD or a co‐occurring late‐onset psychotic syndrome.

Several neuropathological studies have shown a significantly higher proportion of neurodegenerative diseases in VLOSLP. In particular, tau fibrillary tangle [40], LBD and argyrophilic grain disease (AGD) [41] have been reported in previous studies. In the latter study, three out of four patients with AGD and two out of nine patients with LBD did not show dementia even at the final stage. The onset of psychiatric symptoms may precede the onset of dementia due to those diseases by a considerable amount of time, which is consistent with the study by Stafford et al. in which VLOSLP patients had a higher rate of transition to dementia 20 years after initial diagnosis, compared to controls [8]. Some biomarker‐negative participants may therefore have had underlying neurodegenerative pathologies that were not captured by the available assessments, including tauopathies. However, even when neurodegenerative pathology is present, it may not fully explain the emergence of psychosis. Psychosocial factors, including perceived rejection, impaired autonomy, social isolation, sensory impairment, and living alone, may also contribute to the onset of late‐life psychosis [1, 42, 43].

## 5 Limitations

The present study has several limitations. First, the sample size was small, which limited statistical power and precluded multivariable adjustment for potential confounders such as age, age at onset, and baseline cognitive performance. This small sample size may have increased the risk of both type I and type II errors, particularly given the multiple statistical comparisons performed without correction. However, because this was an exploratory study, we prioritised avoiding type II error and interpreted the findings cautiously. Recruiting a sufficient number of patients with VLOSLP was challenging, as these patients often have limited insight into their psychotic symptoms and may disengage from clinical services [44]. Despite our best efforts to work with local dementia outreach teams to identify potential candidates [45], only a limited number of eligible participants had both biomarker and longitudinal follow‐up data. Second, the retrospective design introduced potential selection bias, as the analysed participants represented a subset of the original VLOSLP cohort with available biomarker and follow‐up data. Third, the timing of biomarker assessments and follow‐up evaluations was not uniform across participants. Although we used the available clinical data within a prespecified observation window, prospective studies with standardised biomarker assessment and regular longitudinal follow‐up are needed to confirm these findings. Fourth, baseline clinical differences between the groups may also have contributed to the observed longitudinal outcomes. The BMs‐pos group had a later age at onset, was older at baseline, and had lower baseline cognitive scores. Because the small sample size precluded reliable multivariable adjustment, biomarker status cannot be interpreted as an independent predictor of dementia progression. Finally, progression to dementia was defined operationally using global CDR scores, and dementia subtype diagnoses were based on retrospective chart review. Therefore, diagnostic misclassification cannot be excluded.

## 6 Conclusions

Although this study did not identify a statistically significant difference in progression to dementia between the two groups, the findings suggest that VLOSLP patients who are positive for AD and/or LBD biomarkers may be more likely to be diagnosed with dementia within approximately 2 years than biomarker‐negative patients. These findings support the view that VLOSLP is a clinically defined but biologically heterogeneous syndrome. Larger prospective studies with standardised biomarker assessment and regular longitudinal follow‐up are needed.

## Funding

This work was supported by JSPS KAKENHI Grant Number TK21K15730 and the SENSHIN Medical Research Foundation Overseas Study grant. The funders had no role in the design of the study; collection, analysis, or interpretation of data; or writing of the manuscript.

## Ethics Statement

This retrospective observational study was approved by the Ethical Review Board of the University of Osaka Hospital (Identification number: 19117(T1)). The requirement for written informed consent was waived by the review board, and an opt‐out approach was implemented. All procedures complied with the Declaration of Helsinki and subsequent updates.

## Consent

The manuscript contains anonymised clinical information without directly identifying personal information. The requirement for written informed consent was waived by the review board, and an opt‐out approach was implemented.

## Conflicts of Interest

The authors declare no conflicts of interest.

## Supporting information


**Table S1:** Demographic data of the participants and the unenrolled patients.
**Table S2:** Details of biomarker results and symptoms of participants.

## Data Availability

The data that support the findings of this study are available from the corresponding author upon reasonable request.
