# Supplementary material for: Progression to Dementia in Very Late‐Onset Schizophrenia‐Like Psychosis Stratified by Alzheimer's Disease and Lewy Body Disease Biomarkers: A Retrospective Cohort Study
Source: Psychogeriatrics. 2026 Aug 10;26(5):e70205. doi: 10.1111/psyg.70205 (PMC13457114; doi:10.1111/psyg.70205)
Supplement: Supplementary file 1 [file PSYG-26-0-s001.pdf]

Supplementary Table 1. Demographic data of the participants and the unenrolled patients with VLOSLP

	Unenrolled VLOSLP (n=45)	Participants (n=22)	U	p	r
Age, years	80 (75–84)	81.5 (70.25–85)	510.5	0.841	0.03
Sex, n of female	39 (86.7%)	19 (86.4%)	NA	1.000	NA
Onset age, years	77 (72–81)	75.5 (66.5–82.75)	516.5	0.779	0.04
Education, years	12 (11–12)	12 (12–14)	403.5	0.189	0.15
MMSE	26 (25–27)	26.5 (25–28)	462.0	0.660	0.05
CDR	0.5 (0.5–0.5)	0.5 (0.5–0.5)	485.0	0.811	0.02
CDR-SB	2.5 (1.5–3)	2.5 (1.5–3)	540.0	0.549	0.07
cases with AD biomarkers, n	13	17	NA	NA	NA
positive cases, n	5	8	NA	0.721	NA
cases with LBD biomarkers, n	26	21	NA	NA	NA
positive cases, n	4	7	NA	0.181	NA
followed up cases, n	5	22	NA	NA	NA

Data are presented as median (interquartile range), n (%), or number. The Mann–Whitney U test was used for continuous and ordinal variables, and Fisher's exact test was used for categorical variables. A p-value of less than 0.05 indicated a significant difference in a two-tailed test. Abbreviations: VLOSLP, very-late-onset schizophrenia-like psychosis; MMSE, Mini-Mental State Examination; CDR, Clinical Dementia Rating; AD, Alzheimer's disease; LBD, Lewy body disease; NA, not applicable.

Supplementary Table 2. Details of biomarker results and symptoms of participants

Group	No.	Age	Onset	Sex	AD biomarker	LBD biomarker	CF	VH	RBD	Pism	Dementia type after progression	Psychotic symptoms
BMs-neg	1	64	61	F	PET (-)	MIBG (-)	-	-	-	-	No progression	PD: Her colleagues in her office are spying on her and harassing her over the airwaves. Even strangers are watching her.
	2	67	65	F	PET (-)	FP-CIT (-)	+	+	-	-	No progression	PD, AH: a strange monk living upstairs threatened her; something white sometimes appeared in front of her
	3	68	65	F	PET (-), CSF p-tau (-)	MIBG (-)	+	-	-	-	CDR-defined progression; no specific dementia subtype assigned	VH: a cockroach was in a spaghetti; a spider was in a cup of coffee
	4	72	68	F	CSF p-tau (-)	MIBG (-)	-	-	-	-	No progression	PD, AH: a neighbor living upstairs makes annoying noises (previously, she thought the neighbor was harassing and targeting her with lasers, and she was admitted to other hospital in two years, but her psychosis improved through the changing medications)
	5	81	66	F	PET (-), CSF p-tau (-)	FP-CIT (-)	-	-	-	-	No progression	PD: a neighbor living upstairs spied on her
	6	82	60	F	PET (-), CSF p-tau (-)	MIBG (-)	-	-	-	-	No progression	PD: neighbors bad-mouthed her; she believed she could negotiate with the city office to create a new airport in her town
	7	82	82	F	PET (-)	FP-CIT (-)	-	-	-	-	No progression	PD: neighbors bad-mouthed her
BMs-pos	8	68	68	F	PET (+), CSF p-tau (+)	FP-CIT (-)	-	-	-	-	AD Dementia	PD: unspecified neighbors intruded into her house, moved her belongings, followed her out of her house, and cast lights on her
	9	70	68	F	PET (+)	MIBG (-)	-	-	-	-	AD Dementia	PD: Items from a self-employed beauty salon are stolen by a thief; neighbors tried to break her salon; post office took her savings
	10	79	78	F	PET (+), CSF p-tau (+)	Not assessed	-	-	-	-	AD Dementia	PD: house cleaners steal things from her home.

	11	84	84	F	CSF p-tau (+)	MIBG (-)	-	+	-	-	No progression	PD, AH: unknown male and female staying beyond her house's wall threatened her
	12	85	82	F	PET (+), CSF p-tau (+)	MIBG (-)	-	-	-	-	No progression	PD, AH: her sister-in-law living in neighborhood intruded into her house and steals things; the intruders made noises
	13	86	83	M	PET(+), CSF p-tau (+)	FP-CIT (-), MIBG (-)	-	-	-	-	AD Dementia	PD, AH: Unknown male and female living upstairs observed and threatened him
	14	86	83	F	CSF p-tau (+)	FP-CIT (-)	+	-	-	-	AD Dementia	PD: her eldest son was entering without permission and stealing money and goods, and withdrawing money from her account
	15	66	64	M	Not assessed	FP-CIT (+)	+	+	+	-	DLB	PD, AH, VH: visual hallucinations of strangers and black shadows; strangers ransacked his house
	16	74	73	F	Not assessed	FP-CIT (+)	-	-	+	-	DLB	PD: Her children thought that she was poor; someone upstairs stalked her
	17	77	68	F	CSF p-tau (-)	FP-CIT (+)	-	-	-	-	possible iNPH	PD: someone spied on her, was entering her home
	18	82	81	F	CSF p-tau (-)	FP-CIT (+)	-	-	-	-	No progression	PD: Her husband's affair partner intruded into home, stole things, moved things from place to place, ironed clothes.
	19	85	82	F	Not assessed	FP-CIT (+)	+	-	-	-	No progression	SH: odd sense in her oral cavity, such as a bitter taste, foam coming out of her mouth, or a feeling of a film
	20	87	86	F	CSF p-tau (+)	FP-CIT (-)	-	-	-	-	No progression	PD: cars lying in wait for her; burglars entering her home.
	21	88	85	F	Not assessed	FP-CIT (+), MIBG (+)	+	+	+	-	No progression	VH: visual hallucinations of her grandchild
	22	90	90	M	Not assessed	FP-CIT (+)	+	+	-	-	No progression	PD, AH: hearing music and song continuation; distant people plotting to attack him; quiet speech being overheard; receiving money by going to kindergarten.

Meaning and Abbreviations: For biomarker assessments, (+) and (-) indicate positive and negative results, respectively; “not assessed” indicates that the

biomarker assessment was not performed. For clinical features, + and - indicate presence and absence, respectively. BMs-neg, biomarkers-negative group; BMs-pos, biomarker-positive group; F, female; M, male; AD, Alzheimer's disease; LBD, Lewy body disease; CF, cognitive fluctuation; VH, visual hallucination; RBD, REM sleep behavior disorder; Pism, parkinsonism; PET, amyloid positron emission tomography; CSF, cerebrospinal fluid; FP-CIT, FP-CIT SPECT; MIBG, MIBG myocardial scintigraphy; DLB, dementia with Lewy bodies; iNPH, idiopathic normal pressure hydrocephalus; PD, persecutory delusions; AH, auditory hallucinations; VH, visual hallucinations; SH, somatic hallucination.
